# Generation of Zucchini Tigre Mosaic Virus Mild Strains for Application in Cross-Protection

**DOI:** 10.3390/v18040411

**Published:** 2026-03-26

**Authors:** Chung-Hao Huang, Li-Cheng Chuang, Yuh-Kun Chen

**Affiliations:** 1 Department of Plant Pathology, National Chung Hsing University, Taichung 402, Taiwan; chunghao@nchu.edu.tw (C.-H.H.);; 2 International Master Program in Plant and Microbial Biology, National Chung Hsing University, Taichung 402, Taiwan

**Keywords:** zucchini tigre mosaic virus, HC-Pro, cross-protection, *Cucurbita* species

## Abstract

Zucchini tigre mosaic virus (ZTMV; *Potyvirus pepotigris*), which infects wax gourd (*Benincasa hispida*), was first identified in Taiwan in 2017 and designated ZTMV-TW. In this study, mild strains of ZTMV-TW were generated by modifying the pathogenicity factor HC-Pro to develop cross-protection strategies for cucurbit crops. A full-length infectious cDNA clone of ZTMV-TW was cloned in pCAMBIA1304 under the control of the CaMV 35S promoter (ZTMV-TWic). ZTMV-TWic induced typical potyvirus particles, cytoplasmic inclusion bodies, and severe symptoms in wax gourd, pumpkin, and zucchini plants. Conserved motifs of HC-Pro were mutated to generate four single mutants (F7I, R181I, F206L, and D397N) and three double mutants (F7I+F206L, R181I+D397N, and F206L+D397N). Mutants R181I and R181I+D397N caused mild or no symptoms in zucchini, while D397N and F206L+D397N were mild in wax gourd. Cross-protection assays showed that R181I and R181I+D397N provided complete protection against ZTMV-GFP in zucchini, whereas D397N and F206L+D397N conferred high protection in wax gourd. These results demonstrate the feasibility of host-specific mild strain selection for effective ZTMV cross-protection.

## 1. Introduction

The Cucurbitaceae family is one of the most important families of crops worldwide, including a wide variety of vegetables and fruits. According to statistics from the Food and Agriculture Organization (FAO) in 2021, watermelon (*Citrullus lanatus* (Thunb.) Matsum. & Nakai), cucumber (*Cucumis sativus* L.), pumpkin (*Cucurbita moschata* Duchesne ex Poir.), and melon (*Cucumis melo* L.) are the four major cultivated cucurbit crops worldwide (FAO, 2021; accessed 25 July 2023). In Taiwan, the diversity of cultivated cucurbits is rich, including not only the four aforementioned species but also loofah (*Luffa aegyptiaca* Mill.), bitter gourd (*Momordica charantia* L.), and wax gourd (*Benincasa hispida* (Thunb.)). Among these, wax gourd and zucchini (or summer squash, *Cucurbita pepo* var. *cylindrica*) have been cultivated in Taiwan for many years. Over the past few decades, the number of viruses infecting cucurbit plants has increased dramatically, a phenomenon believed to be associated with global warming and climate change [1]. To date, at least 90 cucurbit-infecting viruses have been reported globally [2], and more than 18 virus species have been identified in Taiwan [3,4,5]. In 2017, a potyvirus was isolated from wax gourd plants in Changhua County, Taiwan, and subsequent genomic and molecular analyses confirmed the first occurrence of zucchini tigre mosaic virus in Taiwan (ZTMV-TW) [3]. This represents an emerging and a potential viral threat to cucurbit crops in Taiwan.

Management of cucurbit viral diseases has relied mainly on field sanitation, the use of virus-free seedlings, and insecticides for controlling insect vectors [1]. Although traditional breeding confers resistance to virus diseases, the identification of resistance genes and the development of resistant cultivars are time-consuming and labor-intensive processes. While genetic engineering can overcome these limitations, public acceptance of genetically modified crops remains limited [6].

Cross-protection provides a non-transgenic strategy for managing viral diseases and has long been applied in practice. The concept is that infection by a mild strain of a virus can protect a plant from subsequent infection by a sequence-homologous severe strain [6,7,8]. Cross-protection proceeds through several interconnected phases. Initially, the mild (protective) strain virus accumulates and triggers the host’s post-transcriptional gene silencing (PTGS) defense. Subsequently, the virus reduces its ability to suppress PTGS, allowing the highly active silencing machinery from the host to effectively target and inhibit the invading severe strain in a sequence-dependent manner. The establishment of complete protections maintained as the protective virus persists at low, fluctuating (“zigzag”) levels. At the same time, protein-mediated resistance occurs through a dominant-negative effect. The proteins of the mild virus interfere with or sequester host factors required for replication and movement of the severe (challenge) strain virus. Moreover, the protective infection also activates the host innate immune responses, such as the salicylic acid (SA)-dependent defense pathway, providing an additional basal layer of resistance [9,10].

Cross-protection has been successfully applied in the field to control viral diseases. Approximately 36 mild viral strains have been reported to confer cross-protection [11]. Among them, citrus tristeza virus (CTV) [12], zucchini yellow mosaic virus (ZYMV) [13], and papaya ringspot virus (PRSV) [14,15,16] have been achieved in commercial cultivation [11]. Other successful cases include applications to PRSV isolates from Vietnam and Taiwan [17,18,19]. ZYMV has caused severe economic losses to the global cucurbit industry. One of the reasons is that resistance genes are few in cucurbits, leading to difficulty of breeding for resistance [20]. A naturally isolated attenuated ZYMV strain (ZYMV-WK) from field-grown zucchini in France was applied in field trials for cross-protection [12]. Moreover, ZYMV-WK was used for cross-protection against ZYMV on cucumber, muskmelon, and zucchini in southern Taiwan [14]. In both studies, plants pre-inoculated with the mild strain were protected from severe ZYMV infection without yield reduction. The mild ZYMV strain was later successfully applied in the United States, Italy, and Turkey [21,22,23]. Recent studies also showed that passion fruit viruses, East Asian passiflora virus (EAPV) [24,25], and passiflora mottle virus (PaMoV) [26] could be controlled using cross-protection strategies. Thus, a mechanical inoculation system for efficient field application was subsequently developed [27].

Among the various functional proteins encoded by potyviruses, HC-Pro (helper component protease) is one of the well-studied and multifunctional proteins. Its N-terminal region is involved in aphid transmission and viral replication [28,29]; the C-terminal region is responsible for protease activity [30]; and the central region contributes to systemic movement and RNA binding and acts as the major PTGS-suppressing function [28,31,32]. Previous studies have demonstrated that mutations in three highly conserved motifs of ZYMV HC-Pro Arg180, Phe206, and Asp396 result in attenuated symptoms and confer effective cross-protection [9,33]. Similarly, in turnip mosaic virus (TuMV) and PRSV, a Phe7 to Ile substitution in the conserved FWKG motif of the N-terminal region reduces symptom expression and confers strong cross-protection [18,34]. Recently, a zucchini tigre mosaic virus (ZTMV-TW) was first identified from wax gourd plants in Taiwan [3]. Given its threat to *Cucurbita* species, it is crucial to develop effective control strategies. However, current management of cucurbit viruses still relies mainly on conventional approaches, and the lack of resistant cultivars poses a significant limitation.

In this study, an infectious clone of ZTMV (ZTMVic) was constructed for subsequent analyses. Based on previous findings [9,17,18,24,25,35], site-directed mutations were introduced at four highly conserved residues within the HC-Pro protein associated with potyvirus pathogenicity to generate mild ZTMV strains with attenuated virulence. These mutants were evaluated for infectivity, symptom expression, viral accumulation, and cross-protection efficacy to identify potential mild ZTMV strains suitable for cross-protection in cucurbit crops. Our results reveal that ZTMV mild strains provided high degrees of cross-protection against a severe ZTMV strain in wax gourd and zucchini plants at different protective levels.

## 2. Materials and Methods

### 2.1. Virus Source

The zucchini tigre mosaic virus Taiwan isolate (ZTMV-TW) (GenBank LC371337) was collected from wax gourd (*Benincasa hispida*) in Changhua County, Taiwan [3,4]. Leaf tissue from ZTMV-TW-infected plants was ground in phosphate buffer (0.1 M phosphate buffer, pH 7.0) at a ratio of 1:10 (*w*/*v*). The crude sap was mechanically inoculated onto the host plant, summer squash (*Cucurbita pepo* var. *cylindrica*). Inoculated plants were grown in a temperature-controlled greenhouse (25 ± 3 °C) for subsequent research.

### 2.2. Construction of ZTMV Infectious Clone (p35S ZTMV-TW)

Total RNA was extracted from leaves of ZTMV-TW-infected summer squash plants using the GENEzolTM TriRNA Pure Kit (Geneaid, Taipei, Taiwan) according to the manufacturer’s instructions. ZTMV-TW three partial cDNAs, which cover the full-length ZTMV-TW RNA genome with overlapping sequence, were reverse-transcribed using SuperscriptTM III Reverse Transcriptase (Invitrogen, Carlsbad, CA, USA) with ZTMV-specific primer or Oligo-dT (pC-ZTMV-1R, pV-ZTMV-II R, and SnaBI-Oligo-dT) (Table S1). Three ZTMV-TW DNA fragments were amplified using individual cDNAs as templates by Phusion^®^ High-Fidelity DNA Polymerase (Thermo Fisher Scientific, Waltham, MA, USA) with three sets of ZTMV-specific primers (pC-ZTMV-OE-F/pC-ZTMV-1R, pC-ZTMV-2F/pV-ZTMV-II R, and pV-ZTMV-II F/SnaBI-Oligo-dT) (Table S1). This process generated three DNA fragments, I, II, and III, with overlapping sequences that cover the entire viral genome (Figure S1). The expected sizes of the amplified cDNA fragments I, II, and III were 3438 bp, 3412 bp, and 3518 bp, respectively. The p35S promoter sequence of plasmid pCAMBIA1304 was used as a template and amplified by PCR with the specific primer pair (pC-p35S-F/pC-p35S-OE-R) (Table S1) to generate a 35S promoter DNA fragment. The 35S promoter DNA fragment and “Fragment I” were ligated and amplified via overlapping PCR with the primer pair (pC-p35S-F/pC-ZTMV-1R) (Table S1) to generate 35S promoter + Fragment I DNA fragment. Then, the fragment was cloned into pCAMBIA1304 Plant Expression Vector (Abcam, Cambridge, UK), which contains the cauliflower mosaic virus (CaMV) 35S promoter and the NOS (nopaline synthase) terminator with *Xba*I and *Nco*I to generate pCAMBIA1304+Fragment I. Fragment II and Fragment III were cloned into pCAMBIA1304+Fragment I with *Nco*I, *SnaB*I, and *NgoM*IV sequentially to generate the full-length ZTMV-TW cDNA infectious clone, named p35S ZTMV-TW. The activated virus derived from the clone was denoted ZTMV-TWic and was used in subsequent studies (Figure S1).

### 2.3. Agrobacterium-Mediated Infiltration (Agro-Infiltration)

The *Agrobacterium tumefaciens* strain GV3101 cell, which contains the p35S ZTMV-TW plasmid, was cultured in YEP medium containing 50 μg/mL Kanamycin and Rifampicin, 10 mM MES (4-Morpholineethanesulfonic acid) pH 5.6, and 0.5 mM acetosyringone (AS) at 28 °C until O.D_600nm_ reached 0.8. The culture was centrifuged to pellet the agrobacteria cells, and the pellet was resuspended in infiltration buffer (10 mM MgCl_2_ and 0.5 mM AS). The culture was then incubated at room temperature for 3 h before infiltration.

### 2.4. Negative Staining and Ultrathin Section

The negative staining was used to observe virus particles. Diseased leaves infected with ZTMV-TW or ZTMV-TWic were cut and mixed in sterile water. A 20 μL aliquot of the crude sap was placed on Parafilm, and a copper grid was immersed in the crude sap for 1–5 min. The copper grid was rinsed with sterile water and then immersed in a 2% aqueous solution of uranyl acetate (UA) for 1–3 min. The sample was observed by a transmission electron microscope (TEM) (JEM-1400, JEOL, Tokyo, Japan). The ZTMV-TW or ZTMV-TWic-infected leaves was fixed in 2.5% glutaraldehyde at 4 °C for 2–4 h and washed four times (15–20 min each) with phosphate buffer (0.1 M phosphate buffer, pH 7.0). Post-fixation was performed with 2% osmium tetroxide (OsO_4_) at 4 °C for 1–2 h, followed by four washes with phosphate buffer (5–10 min each). The tissue was sequentially dehydrated with an ethanol series (50%, 70%, 85%, 95%, and 100%). Subsequently, the tissue was immersed in a 50% LR White Resin-100% EtOH mixture (*v*/*v* = 1:1) overnight at 4 °C, followed by immersion in 100% LR White Resin overnight at 4 °C. Finally, the sample was embedded in capsules until the resin hardened. Ultrathin sections of approximately 70–90 nm were cut using an ultra-microtome (Leica UC-7, Wetzlar, Germany), followed by double staining with uranyl acetate and lead citrate [36]. After washing with distilled water, the section was observed with the TEM.

### 2.5. Indirect Enzyme-Linked Immunosorbent Assay (Indirect ELISA)

The samples were collected from tissue infected with ZTMV-TW or individual ZTMV mutants. A total of nine leaf discs (0.5 cm in diameter) were taken from three different upper leaves (three discs, around 50 mg, from the first fully expanded leaf and three discs each from the leaf immediately above and below it). The discs from a single plant were combined into a single sample (three plants pooled as one replicate). Crude sap was extracted from the tissue sample described above using coating buffer (15 mM Na_2_CO_3_, 35 mM NaHCO_3_, 3.1 mM NaN_3_, pH 9.6) at a ratio of 1:200 (*w*/*v*). A 100 μL aliquot was added to a 96-well plate and incubated at 37 °C for 1 h (one biological replicate measured with three technical replicates in different wells). After washing three times with PBST (phosphate-buffered saline, pH 7.4, containing 0.05% Tween 20) for 3 min each, 100 μL of ZTMV-TW antiserum [3] diluted 1:5000 was added, and the plate was incubated at 37 °C for 1 h. The plate was washed three times with PBST for 3 min each. A 100 μL of secondary antibody (alkaline phosphatase conjugated goat anti-rabbit IgG, Invitrogen) diluted 1:5000 was added, followed by incubation at 37 °C for 1 h. The plate was washed three times with PBST for 3 min each. An amount of 100 μL of AP substrate (1 mg/mL p-nitrophenyl phosphate, PNPP) dissolved in substrate buffer (9.7% diethanolamide and 0.02% NaN_3_, pH 9.8) was added. The absorbance (405 nm) was measured using a microplate reader (Multisken FC, Thermo, Taichuang, Taiwan) [37].

### 2.6. Western Blotting

Plant tissue was ground in 5X protein sample buffer (5% SDS, 50% glycerol, 0.05% bromophenol blue, and 25% *β*-mercaptoethanol), and the extract was heated at 95 °C. Then, the supernatant was collected after centrifugation (10,000× *g*, 3 min). The proteins were resolved on a 12% SDS (sodium dodecyl sulfate-polyacrylamide) gel and transferred to a PVDF membrane (PerkinElmer, Waltham, MA, USA). After transfer, the membrane was immersed in TSW blocking solution (10 mM Tris, 0.9% NaCl, 5% non-fat milk, 0.1% Triton X-100, 0.02% SDS) and shaken at room temperature for 1 h. ZTMV-TW antiserum [3] diluted 1:5000 was added, and the membrane was shaken at room temperature for 1 h. The membrane was washed three times with TSW (10 mM Tris, 0.9% NaCl, 3% non-fat milk, 0.1% Triton × 100, 0.02% SDS) for 5 min each. Subsequently, secondary antibody (AP conjugated goat anti-rabbit IgG, Invitrogen) diluted 1:5000 was added, and the membrane was shaken at room temperature for 1 h. After washing the PVDF membrane three times with TSW for 10 min each, the Clarity Western ECL substrate (BIORAD) was added for color development. Then, the membrane was stained with Coomassie Brilliant Blue R250 (CBB), and the large subunit of ribulose-1,5-bisphosphate carboxylase/oxygenase (RuBisCO) was used as a loading control.

### 2.7. Construction of ZTMV-GFP Infectious Clone

The DNA fragments, “p35S+P1” (2093 bp) and “HC-Pro+P3” (1733 bp), were amplified with the primer pairs pC-p35S-F/GFP-1R and GFP HC-4F/pC-ZTMV-1R, respectively (Table S1), using the p35S ZTMV-TW plasmid as a template by PCR. The GFP gene fragment (749 bp) was amplified with the primer pair GFP-2F/GFP HC-3R (Table S1) using the pCAMBIA1304 plasmid as a template by PCR. The “p35S+P1+GFP+P3” fragment was amplified with the primer pair pC-p35S-F/pC-ZTMV-1R (Table S1) using “p35S+P1”, “GFP”, and “HC-Pro+P3” fragments by overlapping PCR. The “p35S+P1+GFP+P3” fragment was replaced with the region of p35S+Fragment I in p35S ZTMV-TW vector with *Xba*I and *Nco*I to generate pZTMV-GFP infectious clone. The activated recombinant virus was designated ZTMV-GFP and used in subsequent research.

### 2.8. Generate ZTMV Mutants by Modification of the HC-Pro Gene

The “p35S+Fragment I” DNA fragment, including HC-Pro region, was amplified with the primer pairs pC-p35S-F/pC-ZTMV-1R using p35S ZTMV-TW plasmid as a template by PCR. Then, the fragment was cloned into the plasmid pCR-Blunt II-TOPO (Invitrogen) to generate pCR-Blunt II-p35S-Fragment I. Specific single or double mutations on the HC-Pro gene, F7I, R181I, F206L, D397N, F7I/F206L, R181I/D397N, and F206L/D397N, were made with four sets of specific primer pairs (RHC F7I/FHC F7I, RHC R181I/FHC R181I, RHC F206L/FHC F206L, and RHC D397N/FHC D397N) (Table S1) by site-directed mutagenesis [25]. The positions of the mutation sites are listed in Figure S2. The HC-Pro-mutated p35S-Fragment I fragments were released from the pCR-Blunt II-p35S-Fragment I mutant vectors carrying mutations in the HC-Pro region; then, these fragments were replaced with the p35S+Fragment I region in p35S ZTMV-TW with *Xba*I and *Nco*I to generate pZTMV-HC-Pro mutants.

### 2.9. Time-Course of Accumulation of ZTMV Mutant by ELISA

The accumulation of virus mutants in summer squash (*C. pepo* var. *cylindrica*) and wax gourd (*Benincasa hispida*) plants after inoculation of individual mutants was measured by indirect ELISA using ZTMV-TW antiserum. The samples were collected from individual plants every 3 days until 30 days post-inoculation. A total of nine leaf discs (0.5 cm in diameter) were taken from three different upper leaves (three discs, around 50 mg, from the first fully expanded leaf and three discs each from the leaf immediately above and below it). The discs from a single plant were combined into a single sample (three plants combined as one replicate).

### 2.10. Cross-Protection Evaluation of the ZTMV Mild Strains in Summer Squash and Wax Gourd Plants

The ability to protect summer squash and wax gourd plants against the severe virus ZTMV or ZTMV-TW was investigated. The screened ZTMV mild-symptom virus strains were used as protective viruses, while wild-type ZTMV (ZTMV-TW) or the recombinant virus ZTMV-GFP were used as challenge viruses. Summer squash and wax gourd plants were mechanically inoculated with a protective inoculum comprising attenuated mutants of ZTMV F7I, R181I, F206L, D397N, F7I/F206L, R181I/D397N, and F206L/D397N. After fifteen days, the top two fully expanded leaves of plants were mechanically inoculated with challenge viruses, which were prepared from diseased leaves infected with wild-type ZTMV or ZTMV-GFP using phosphate buffer (pH 7.2) at a ratio of 1:10 (*w*/*v*). The protective efficacy was evaluated based on symptom development in the inoculated plants and the expression of GFP. The presence of the challenge virus was detected by RT-PCR 14 days post-challenge inoculation. Total RNA extracted from inoculated leaf tissue, ZTMV-GFP, and ZTMV mild mutant cDNAs were reverse transcribed using SuperscriptTM III Reverse Transcriptase (Invitrogen, USA) with Oligo-dT. ZTMV partial P3, 6K1 and partial CI gene fragment and GFP gene DNA fragments were amplified by Phusion^®^ High-Fidelity DNA Polymerase (Thermo Scientific) with ZTMV-degenerate primers (fZTMV-F and gourd-R) [4] and GFP-specific primers (GFP-1R and GFP-2F), respectively (Table S1).

### 2.11. Prediction of Protein Tertiary Structure

The amino acid sequences of HC-Pro of ZTMV (GenBank: LC371337), PRSV (GenBank: X97251), TuMV (GenBank: AF530055), and ZYMV (GenBank: AF127929) were collected from the NCBI database. The AlphaFold 3 [38] was used to predict the tertiary structure of HC-Pro proteins. Predicted structural models were generated with default parameters, and the highest-ranking structure was visualized using ChimeraX software V1.10 [39]. Predicted template modeling (pTM) scores were presented as a measure of superposition between the predicted structure and proper structure. A pTM score above 0.5 indicates the overall predicted global fold of the complex is likely to be the true structure.

## 3. Results

### 3.1. Construction of ZTMV-TW Infectious Clone

**Figure 1:**
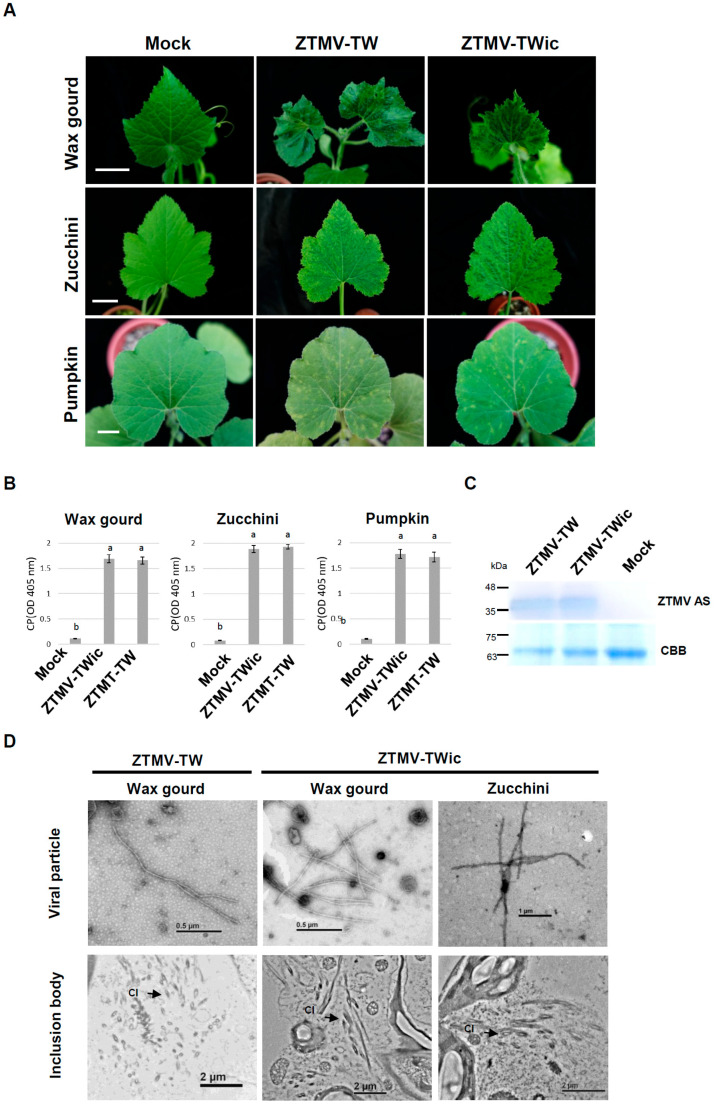
Analysis of infectivity of the in vivo infectious clone of zucchini tigre mosaic virus Taiwan isolate (p35S ZTMV-TW). (**A**) Infectivity assay of p35S ZTMV-TW-activated virus designated as ZTMV-TWic. Symptoms induced by ZTMV-TWic and wild-type (WT) ZTMV-TW in wax gourd, zucchini, and pumpkin plants. Curl, deformity, and tabby mosaic symptoms on systemic leaf in wax gourd plants at 21 days post inoculation (dpi). Vein chlorosis and distinct tiger mosaic symptoms on zucchini plants (14 dpi). Systemic yellow spots on pumpkin leaves (14 dpi). The buffer-treated plant (Mock) was used as a negative control. Scale bar: 3 cm. (**B**) Detection of the accumulation of virus titer in ZTMV-TWic or WT ZTMV-TW-inoculated wax gourd, zucchini, or pumpkin plants at 14 dpi with ZTMV-specific antiserum (ZTMV As) by indirect ELISA. Each absorbance value represents the average of three biological replicates. The same letters indicate no significant difference (*p* < 0.05) according to the one-way ANOVA followed by Tukey’s post hoc test. (**C**) Detection of ZTMV on zucchini inoculated by WT ZTMV-TW and ZTMV-TWic at 14 dpi with ZTMV-specific antiserum (ZTMV As) by Western blotting. The buffer-treated plants (Mock) were used as a negative control; Coomassie brilliant blue (CBB) staining total protein as a loading control. (**D**) Electron micrographs of virus particles in the crude sap (**upper panel**) or infected cell (**lower panel**) of wax gourd, zucchini, and pumpkin plants inoculated with ZTMV-TW or ZTMV-TWic. The CI and arrows indicate the typical cytoplasmic inclusions of potyvirus infection.

**Figure 2:**
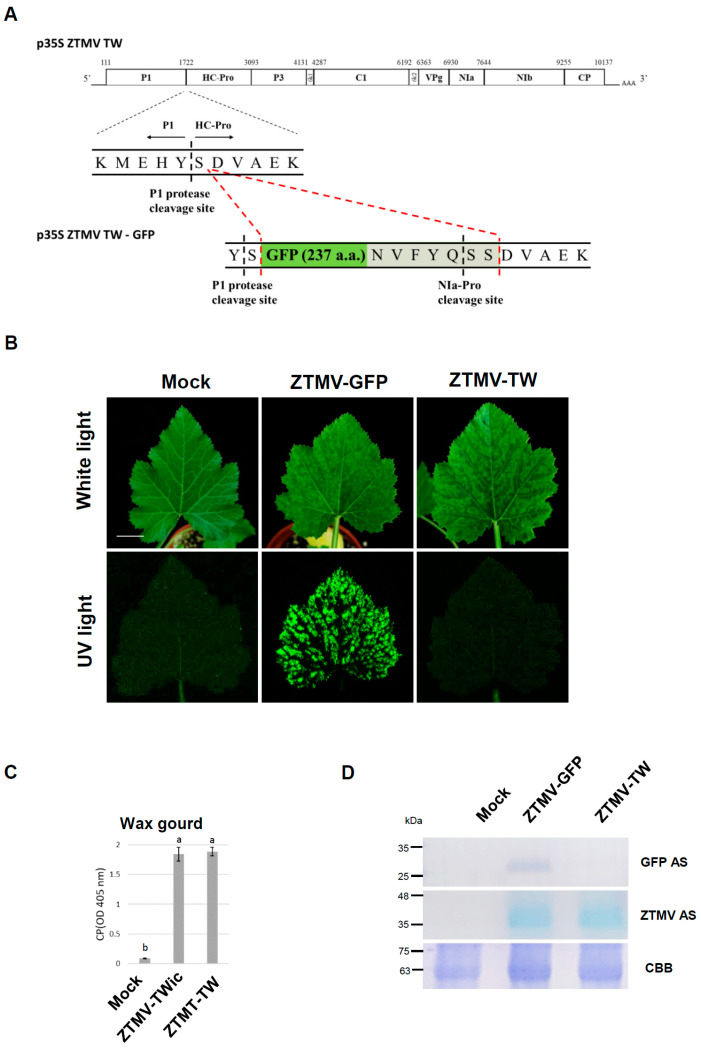
Infectivity assay of zucchini tigre mosaic virus Taiwan isolate carrying green fluorescence protein (GFP) gene (p35S ZTMV-GFP). (**A**) Schematic represents the GFP gene inserted into the ZTMV genome. The GFP sequence was constructed after the 1st amino acid of the HC-Pro sequence (sequence in green), and the C-terminus of the GFP sequence was added with a NIa protease cleavage site (labeled in gray). (**B**) Symptoms of zucchini plants inoculated with the recombinant ZTMV-GFP and ZTMV-TW. Severe systemic mosaic appeared on zucchini plants inoculated with ZTMV-GFP and ZTMV at 14 dpi. Photos were taken under white or UV lights. The buffer-treated plants (Mock) were used as a negative control. Scale bar: 3 cm. (**C**) The accumulation of virus titer in ZTMV-GFP or ZTMV-TW-inoculated zucchini plants at 14 dpi with ZTMV antiserum (ZTMV As) by indirect ELISA. Each absorbance value represents the average of three biological replicates. The same letters indicate no significant difference (*p* < 0.05) according to the one-way ANOVA followed by Tukey’s post hoc test. (**D**) Detection of ZTMV coat protein and GFP on zucchini inoculated by ZTMV-TW and ZTMV-GFP at 14 dpi with ZTMV As and GFP As by Western blotting, respectively. The buffer-treated plants (Mock) were used as a negative control; Coomassie brilliant blue (CBB)-staining total protein as a loading control.

**Figure 3:**
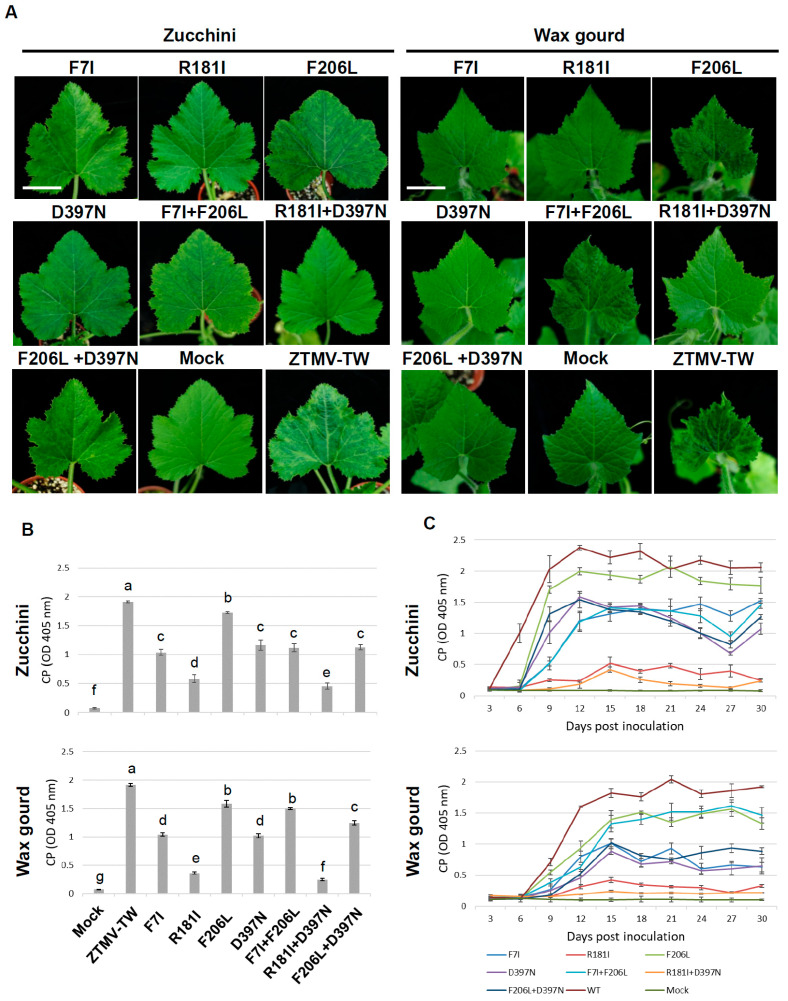
Symptom development of zucchini tigre mosaic virus (ZTMV) mild strains infecting zucchini and wax gourd plants, and time-course accumulation levels of ZTMV coat protein (CP). (**A**) Symptom developed on zucchini (**left panel**) and wax gourd (**right panel**) plants after inoculation with individual ZTMV-TW mutants compared with that induced by the wild-type (WT) ZTMV-TW. Photos were taken at 21 days post-inoculation (dpi). Scale bar: 3 cm. (**B**) The accumulation of virus titer in zucchini (**upper panel**) and wax gourd (**lower panel**) plants inoculated with ZTMV mutants with ZTMV antiserum (ZTMV As) at 14 dpi by indirect ELISA. Each absorbance value represents the average of three biological replicates. The same letters indicate no significant difference (*p* < 0.05) according to the one-way ANOVA followed by Tukey’s post hoc test. (**C**) The time-course of virus accumulation in individual ZTMV mutants infecting zucchini (**upper panel**) and wax gourd (**lower panel**) plants. Each absorbance value represents the average of triplicate readings. Plants inoculated with ZTMV-TW (WT) and buffer (Mock) were used as positive and negative controls, respectively.

The full-length cDNA clone of Taiwan strain ZTMV-TW, designated p35S-ZTMV-TW, was cloned into pCambia1304 between a 35S promoter and a nopaline synthase (nos) terminator by ligating three overlapping cDNA fragments amplified by reverse transcription-PCR (RT-PCR) from viral RNA (Figure S1). The activated virus derived from the ZTMV infectious clone (p35S ZTMV-TW), designated ZTMV-TWic, consistently induced symptoms similar to those caused by the wild-type ZTMV (ZTMV-TW) in three systemic hosts, including wax gourd, zucchini, and pumpkin plants (Figure 1A), confirming that the constructed ZTMV-TW infectious clone was infectious. Indirect ELISA using ZTMV-TW antiserum showed no significant difference in the accumulation of the coat protein (CP) between ZTMV-TWic and wild-type ZTMV in wax gourd, zucchini, and pumpkin plants (Figure 1B,C). Western blot analysis of ZTMV-TWic using ZTMV-TW antiserum detected the 35 kDa ZTMV CP in infected zucchini plants (Figure 1D). Transmission electron microscopy (TEM) examination of ZTMV-TWic-infected wax gourd and zucchini samples revealed filamentous virus particles (Figure 1D, upper panel) and cone-shaped inclusion bodies (Figure 1D, lower panel), which were consistent with typical potyvirus particles and inclusion bodies formed by wild-type ZTMV (Figure 2A and Figure 3A) The results indicated that the artificial construction process did not adversely affect the morphology or pathogenicity of the ZTMV infectious clone.

### 3.2. Infectivity Assays of ZTMV-GFP Infectious Clone

To better monitor virus infection and distinguish the wild-type ZTMV and ZTMV mild mutants in infected plants, the ZTMV-TW carrying an additional gene (ZTMV-GFP) was constructed. The recombinant virus (ZTMV-GFP) activated from the recombinant infectious clone (pZTMV-GFP) caused vein chlorosis and mild mosaic symptoms on systemic leaves of zucchini at 7 dpi, which progressed to a pronounced tigre mosaic pattern by 14 dpi, similar to the symptoms induced by ZTMV-TW infection (Figure 2B, upper panel). Placing the ZTMV-GFP-infected leaves under UV light revealed distinct green fluorescence (Figure 2B, lower panel), confirming that the ZTMV-GFP recombinant virus is infectious and induces symptoms similar to those of ZTMV-TW infection. Indirect ELISA using ZTMV-TW antiserum showed no difference in coat protein (CP) accumulation between ZTMV-GFP and wild-type ZTMV (Figure 2C). Western blot analysis of ZTMV-GFP using ZTMV-TW antiserum and GFP antiserum detected the ZTMV CP and the GFP, respectively, in infected zucchini plants (Figure 2D).

### 3.3. Site-Directed Mutations in the HC-Pro Gene of ZTMV-TW

Site-directed mutagenesis was conducted on the ZTMV infectious clone to generate four single-point mutated infectious clones (mICs) [mICs F7I (Phe7→Ile), R181I (Arg181→Ile), F206L (Phe206→Leu), and D397N (Asp397→Asn)] and three double-point mutated infectious clones [mICs F7I+F206L (Phe7→Ile+Phe206→Leu), F206L+D397N (Phe206→Leu+Asp397→Asn), and R181I+D397N (Arg181→Ile+Asp397→Asn)] (Figure S2). All seven ZTMV mutant infectious clones (pZTMV-mutants) were infectious in the systemic hosts zucchini and wax gourd (Figure 3A). In systemic zucchini plants, the mutant ZTMV-F206L caused vein chlorosis on systemic leaves at 7 dpi, followed by a mosaic at 14 dpi, and a prominent tigre mosaic pattern at 21 dpi, similar to wild-type ZTMV symptoms. Four ZTMV-mutants, F7I, D397N, F7I+F206L, and F206L+D397N, caused a mosaic on systemic leaves at 14 dpi, followed by an attenuated tigre mosaic pattern at 21 dpi. Two ZTMV-mutants, R181I and R181I+D397N, induced only mild and inconspicuous symptoms on systemic leaves (Figure 3A, left panel). The virus accumulation level of ZTMV-mutant F206L was similar to that of the wild-type virus in both zucchini and wax gourd plants at 14 dpi (Figure 3B). The viral titers of ZTMV-mutants F7I, D397N, F7I+F206L, and F206L+D397N were significantly lower than the wild-type virus, whereas ZTMV-mutants R181I and R181I+D397N showed the lowest accumulation among the seven mutants (Figure 3B, upper panel). In systemic wax gourd plants, ZTMV-mutants F206L and F7I+F206L caused crinkling, deformation, and mosaic symptoms on new leaves at 14 dpi, similar to the wild-type ZTMV symptoms. ZTMV-mutants F7I, R181I, D397N, F206L+D397N, and R181I+D397N all caused inconspicuous symptoms on new leaves (Figure 3A, right panel). Viral accumulation of the seven ZTMV mutants was measured at 14 dpi (Figure 3B, lower panel). Analysis revealed that ZTMV mutants F206L and F7I+F206L accumulated less virus than the ZTMV-TW. Furthermore, accumulation was significantly reduced for mutants F7I, D397N, and F206L+D397N compared to the WT virus. Among all seven tested strains, ZTMV mutants R181I and R181I+D397N showed the lowest viral accumulation.

### 3.4. Analysis of the Accumulation Dynamics of ZTMV Mutants

For analyzing the viral accumulation dynamics of ZTMV mutants in zucchini and wax gourd plants, the accumulation of the seven ZTMV mutants was monitored every three days up to 30 dpi in mechanically inoculated plants by indirect ELISA. In zucchini plants, the CP accumulation of ZTMV-mutant F206L was slightly lower than that of wild-type ZTMV but maintained a high accumulation level as the days post-inoculation increased. CP accumulation for ZTMV-mutants F7I and F7I+F206L gradually increased until 15 dpi, after which it remained relatively constant. CP accumulation for ZTMV-mutants D397N and F206L+D397N gradually increased until 12 dpi, followed by a slight decrease until 27 dpi. CP accumulation for ZTMV-mutants, R181I and R181I+D397N, slowly increased until 15 dpi; subsequently, the accumulation of ZTMV-mutant R181I slightly decreased and then increased, exhibiting a zigzag pattern in the dynamic change graph, while the accumulation of ZTMV-mutant R181I+D397N slowly decreased and remained at an extremely low titer (Figure 3C, upper panel). In wax gourd plants, ELISA results showed that the CP accumulation of all seven mutant strains was significantly lower than that of wild-type ZTMV. CP accumulation for ZTMV-mutants F206L and F7I+F206L gradually increased until 15 dpi, after which it was maintained at a high accumulation level. CP accumulation for ZTMV-mutants F7I, D397N, and F206L+D397N increased over time, reaching a first peak at 15 dpi, followed by a slight decrease and then an increase, maintaining a similar accumulation level (Figure 3C, lower panel).

### 3.5. Cross-Protection Effectiveness of Selected ZTMV Mild Strains Against ZTMV-TW and ZTMV-GFP in Zucchini and Wax Gourd Plants

**Figure 4:**
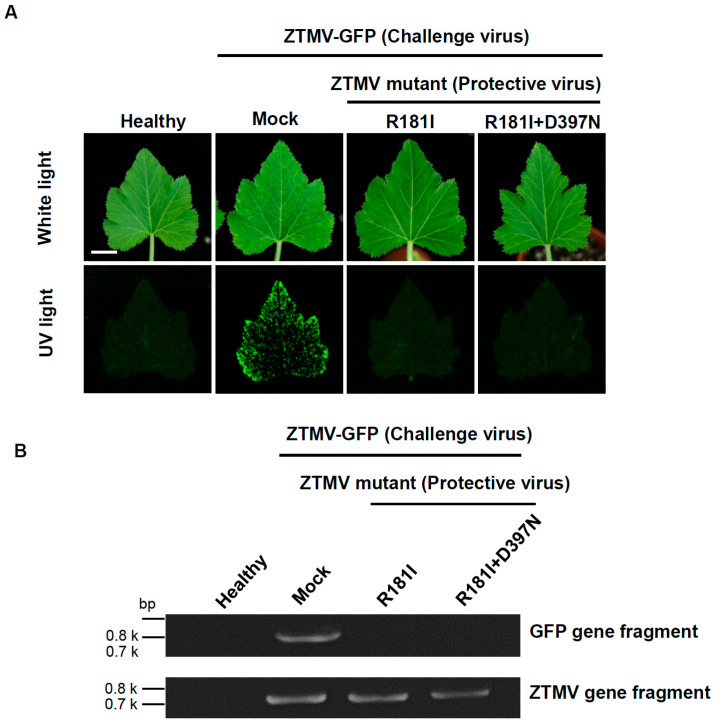
The cross-protection effectiveness of the zucchini tigre mosaic virus (ZTMV) mild viruses R181I and R181I+D397N against the severe recombinant ZTMV-GFP on zucchini plants. All plants were challenged with ZTMV-GFP (challenging virus) 15 days after the inoculation with mild viruses R181I and R181I+D397N (protective virus). (**A**) Symptoms on zucchini plants were photographed under white light and UV light at 14 days after the challenge inoculation with ZTMV-GFP. Scale bar: 3 cm. (**B**) ZTMV gene and GFP gene fragments were detected by RT-PCR using degenerate primers and specific primers for ZTMV and GFP genes. ZTMV-GFP-inoculated plants (Mock) and buffer-treated (Healthy) plants were used as positive and negative controls, respectively.

Based on the infectivity and symptoms on plants of the ZTMV mild strains, the mild strains (protective viruses) R181I and R181I+D397N were selected for cross-protection assays in zucchini plants to analyze the effectiveness against inoculation with ZTMV-GFP (challenge virus). The mild strains used to inoculate the zucchini plants were challenged by mechanical inoculation with the recombinant virus ZTMV-GFP at 14 days after mild strain inoculation. The systemic leaves of zucchini plants protected by mild strains R181I and R181I+D397N showed no symptoms and no green fluorescence under UV light after inoculation with ZTMV-GFP (Figure 4A), indicating that mild strain R181I and R181I+D397N provided excellent cross-protection (100%) against ZTMV-GFP in zucchini plants (Table 1). The ZTMV cDNA fragment was amplified by RT-PCR with the ZTMV gene-specific primers (fZTMV-F/gourd-R) but no GFP gene fragment was amplified with GFP primers (GFP-2F/GFP HC-3R) from ZTMV-GFP-challenged plants (Figure 4B). The results indicated that the plants were successfully protected by the mild strains R181I and R181I+D397N, which prevented ZTMV-GFP infection and subsequent GFP synthesis.

**Figure 5:**
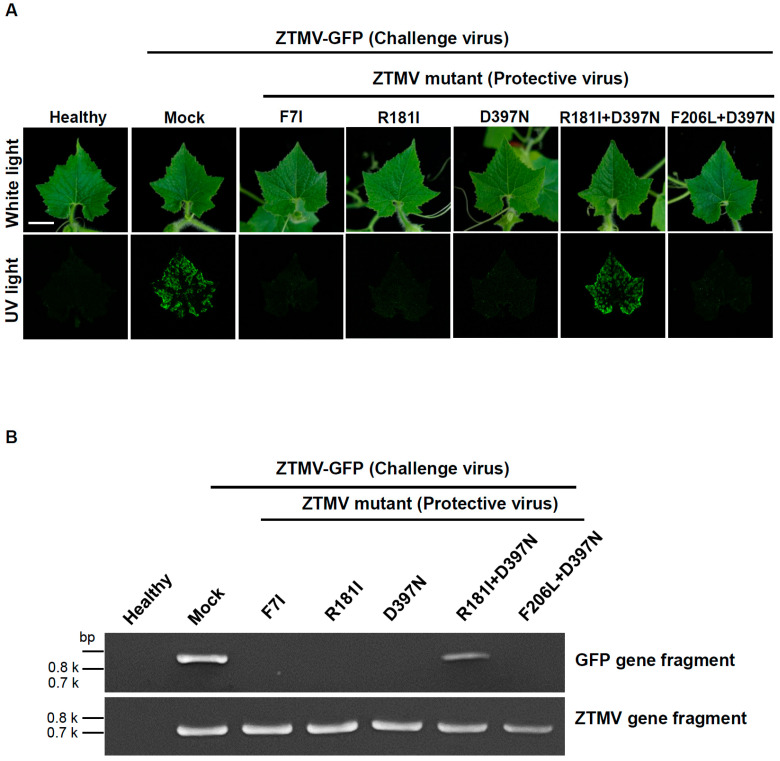
The cross-protection effectiveness of the zucchini tigre mosaic virus (ZTMV) mild viruses F7I, R181I, D397N, R181I+D397N, and F206L+D397N against the severe recombinant ZTMV-GFP on wax gourd plants. All plants were challenged with ZTMV-GFP (challenging virus) 15 days after the inoculation with mild viruses F7I, R181I, D397N, R181I+D397N, and F206L+D397N (protective virus). (**A**) Symptoms on wax gourd plants were photographed under white light and UV light at 14 days after the challenge inoculation with ZTMV-GFP. Scale bar: 3 cm. (**B**) ZTMV gene and GFP gene fragments were detected by RT-PCR using degenerate primers and specific primers for ZTMV and GFP genes. ZTMV-GFP-inoculated plants (Mock) and buffer-treated (Healthy) plants were used as positive and negative controls, respectively.

The five ZTMV mild strains (F7I, R181I, D397N, F206L+D397N, and R181I+D397N) were chosen as protective viruses in wax gourd plants. The systemic leaves of wax gourd plants inoculated with the individual mild strain remained symptomless 14 days after challenge inoculation with ZTMV-GFP (Figure 5A). However, approximately half of the plants protected by R181I+D397N exhibited green fluorescence under UV light (Table 1 and Figure 5A). RT-PCR analysis revealed that the ZTMV cDNA fragments were detected in all mild strain inoculated plants. In contrast, the GFP gene fragment was detected only in R181I+D397N-inoculated plants (Figure 5B), confirming the presence and replication of the recombinant mild strain virus in the plants. The result indicated that while F7I, R181I, D397N, and F206L+D397N conferred complete cross-protection (100%) by interfering with severe strain ZTMV-GFP infection in plants (Table 1), the plants inoculated with R181I+D397N were still partially susceptible to ZTMV-GFP infection (50% infection rate, Table 1), despite showing no visible symptoms (Figure 5A).

In contrast, the cross-protection effectiveness against challenge with wild-type (WT) ZTMV in wax gourd plants varied markedly among the mild strains (Figure S3). The D397N conferred the highest protection (100%), followed by F206L+D397N (94%) and F7I (69%). In comparison, R181I (17%) and R181I+D397N (8%) provided little to no protection, as most of these plants developed clear systemic mosaic symptoms upon WT challenge (Table 1 and Figure S3).

## 4. Discussion

To generate mild strains of ZTMV through molecular biotechnology, we aimed to attenuate its pathogenic determinant, the HC-Pro gene precisely. In this study, we first constructed an infectious cDNA clone of ZTMV, which was capable of infecting various cucurbit hosts (Figure 1). Based on previous studies of potyviruses such as PRSV [17,18,40,41], TuMV [34], East Asian passiflora virus [24,25] and ZYMV [9,33], four conserved amino acid residues within the HC-Pro gene of ZTMV-TW were selected for site-directed mutagenesis, resulting in the substitutions F7I, R181I, F206L, and D397N. The four single mutants caused varying degrees of symptom expression on zucchini and wax gourd plants (Figure 3A). ELISA analyses revealed that their viral accumulation levels at 14 dpi were lower than those of WT ZTMV-TW (Figure 3B). Previous research has shown that the N-terminal highly conserved FWKG motif of TuMV HC-Pro is associated with its RNA-silencing suppressor function through disrupting dimerization [34]. The mild strain F7I caused no visible symptoms on wax gourd plants but produced typical mosaic symptoms on zucchini plants (Figure 3A), suggesting reduced infectivity in wax gourd. The amino acid residues F205 and D396 are also highly conserved in ZYMV HC-Pro and have been shown to affect viral pathogenicity [9,40]. However, the corresponding amino acid position mutation F206L on ZTMV caused severe symptoms in both zucchini and wax gourd plants, whereas D397N induced mosaic symptoms on zucchini but only mild symptoms on wax gourd (Figure 3A). These results indicate that substitution at F206L does not attenuate pathogenicity, whereas mutation at D397N weakens infectivity in zucchini and markedly reduces virulence in wax gourd. The mutant R181I produced no obvious symptoms in either zucchini or wax gourd plants (Figure 3A), suggesting attenuated infectivity in both host plants. The FRNK motif in HC-Pro is highly conserved among potyviruses and is essential for gene silencing suppression [42]. The ability of HC-Pro to bind siRNA duplexes requires the FRNK motif, and substitution of a polar amino acid with a hydrophobic one can interfere with its interaction with host factors [33]. To enhance the genetic stability of mild strains for use in double mutants, combinations of mutations previously reported to reduce viral pathogenicity [9,17,18] were introduced, including F7I+F206L, R181I+D397N, and F206L+D397N. These double mutants exhibited varying infectivity in zucchini and wax gourd plants. All mutants accumulated lower levels of virus titer than WT ZTMV-TW at 14 dpi, with R181I+D397N exhibiting the lowest viral titer in both hosts (Figure 3B). The strain F7I+F206L caused more severe symptoms on wax gourd plants but milder mosaic symptoms on zucchini plants, differing from the reported mild phenotypes [43]. The mutant R181I+D397N caused no visible symptoms in either host, confirming its highly attenuated. Meanwhile, F206L+D397N induced mosaic symptoms on zucchini plants but only mild symptoms on wax gourd plants, indicating greater infectivity in zucchini and weaker pathogenicity in wax gourd plants.

Taken together, we introduced similar mutations at HC-Pro as previous studies, e.g., PRSV [17,18], TuMV [34], ZYMV [9], EAPV [24,25], PaMoV [26], and PLDMV [35], into ZTMV-TW to generate suitable mild strains for evaluation of the cross-protection effectiveness on zucchini and wax gourd plants. Nevertheless, the plants infected with individual mutants showed symptoms of varying degrees (Figure 3A) compared to other potyvirus mild mutants (PRSV, TuMV, ZYMV, EAPV, PaMoV, and PLDMV). For example, the PRSV (F7I and F7I +F206L) [34], ZYMV (R181A+F206L and R181A+D396N) [9], and EAPV (F7I+R181A and R181A+D396N) [25] mild mutants showed mild symptoms and better cross-protection effectiveness. We compare the predicted tertiary structures of each HC-Pro protein, and the results reveal that the C-terminus regions of the HC-Pro proteins are highly similar (Figure S4). So, we assumed that symptoms of varying degrees caused by ZTMV and other mild potyviral strains may be due to interactions between HC-Pro proteins and HC-Pro-interacting host proteins, not the protein structures themselves.

An ideal mild strain for cross-protection should display a zigzag pattern of viral accumulation over time, consisting of initiation, resistance, and maintenance phases [9,10]. During initiation, viral replication rapidly increases; in the resistance phase, host defenses are triggered by viruses; thus, the viral titer is reduced. Finally, in the maintenance phase, a balance is reached between viral replication and host defenses. Based on the time-course of the virus titer results, the viral accumulation of F7I, F206L, D397N, F7I+F206L, and F206L+D397N in zucchini remained lower than that of WT ZTMV-TW (Figure 3C), but all continued to cause visible mosaic symptoms (Figure 3A). Therefore, these strains are not suitable as protective viruses for cross-protection in zucchini plants. In contrast, R181I and R181I+D397N exhibited significantly lower accumulation than ZTMV-TW and other mild strains (Figure 3B) and induced no visible symptoms in zucchini plants (Figure 3A). Furthermore, the time-course patterns of R181I and R181I+D397N mutants showed the typical “zigzag” fluctuation. Both provided effective cross-protection (Table 1 and Figure 4). These results suggest that R181I and R181I+D397N mutants are promising mild strains for cross-protection in zucchini plants. In wax gourd plants, the accumulation of F206L and F7I+F206L was slightly lower than ZTMV-TW (Figure 3B), but both caused severe symptoms (Figure 3A), indicating poor suitability for cross-protection. The mutants F7I, R181I, D397N, R181I+D397N, and F206L+D397N all exhibited significantly lower accumulation and induced no apparent symptoms (Figure 3A). Among them, F7I, R181I, D397N, and F206L+D397N mutants displayed a “zigzag” pattern of virus accumulation, suggesting potential for cross-protection in wax gourd plants. The cross-protection effectiveness of R181I and R181I+D397N mutants was evaluated against both ZTMV-GFP recombinant virus (Figure 5) and ZTMV-TW (Figure S3).

In this study, we investigated the cross-protection efficacy of the mild strains R181I and R181I+D397N against challenge by the ZTMV-GFP recombinant virus and ZTMV-TW. When the mild strains were applied to zucchini plants, both mild strains conferred excellent cross-protection against ZTMV-GFP (Table 1). However, when applied in wax gourd plants against ZTMV-GFP, the mild strain R181I provided better cross-protection, while R181I+D397N showed only 50% effectiveness (Table 1). Furthermore, when R181I and R181I+D397N mild strains were applied for cross-protection against ZTMV-TW in wax gourd plants, most protected plants still developed severe symptoms after challenge inoculation (Figure S3). This result indicated that neither mild strain effectively protected the plants against the homologous severe virus (Table 1). Our findings suggest that R181I and R181I+D397N can successfully establish infection and induce host defense responses to achieve cross-protection in zucchini plants, supporting their preliminary feasibility for application. Nevertheless, the cross-protection capabilities of these two mild strains against ZTMV-TW needed further validation trials for more convincing application. The excellent cross-protection of R181I and R181I+D397N against the recombinant virus ZTMV-GFP in wax gourd plants but failure to protect plant against the homologous ZTMV-TW is hypothesized to be due to their low viral titers in wax gourd plants, as suggested by the time-course accumulation assays (Figure 3C). This low titer likely prevented the induction of sufficient plant immune response, resulting in plants susceptible to the subsequent severe viral infection. We also tested the cross-protection effectiveness of mild strains F7I, D397N, and F206L+D397N in wax gourd plants against both ZTMV-GFP and WT ZTMV-TW. All three strains provided better cross-protection against ZTMV-GFP in wax gourd (Figure 5 and Table 1). Furthermore, D397N also demonstrated superior cross-protection against wild-type ZTMV-TW, while F7I and F206L+D397N still provided moderate and high levels of cross-protection, respectively, against wild-type ZTMV-TW (Table 1). These results indicate that mild strains F7I, D397N, and F206L+D397N can establish infection and induce host defense responses in wax gourd plants, thereby offering effective cross-protection against the homologous severe virus. Notably, the high protection conferred by D397N and F206L+D397N suggests the practical applicability of these two mild strains for cross-protection management of the homologous ZTMV in wax gourd plants.

Previous studies have reported that insertion sites between P1/HC-Pro [43,44] and NIb/CP [45,46] in potyvirus genomes are suitable for introducing marker genes while maintaining infectivity. However, the stability of foreign gene expression varies depending on the insertion site and virus species [47,48,49], showing that inserting GFP at the N-terminus of HC-Pro in TuMV-YC5 resulted in milder symptoms than other insertion sites, and the expression stability of GFP differed among hosts. In this study, GFP was inserted between P1 and HC-Pro in the infectious clone p35S ZTMV-TW (ZTMV-IC) to generate the recombinant virus ZTMV-GFP (Figure 2A). In cross-protection assays, ZTMV-GFP induced typical mosaic symptoms in zucchini (Figure 4A); moreover, GFP expression was observed, and symptoms were absent in wax gourd plant (Figure 5A). Western blotting revealed the accumulation of fused GFP forms in infected wax gourd, indicating inefficient protein cleavage that may impair HC-Pro function and reduce viral pathogenicity. Therefore, ZTMV-GFP represents a weaker virus in wax gourd and is less suitable as a challenge virus for evaluating cross-protection. Consequently, ZTMV-TW was used as the challenge virus in wax gourd plant in subsequent assays. The result revealed that ZTMV mild mutants provided less protection when challenged with ZTMV-TW in wax gourd plant, except D397N (Table 1, Figure S3). Thus, we will use ZTMV-WT as challenge virus for cross-protection assay to evaluate the cross-protection rate in zucchini plants as well.

An ideal mild strain for cross-protection should induce mild systemic symptoms, remain genetically stable, be non-transmissible by insect vectors, and lack synergistic interactions with other viruses [11]. The mild strains F7I, R181I, D397N, R181I+D397N, and F206L+D397N met the first criterion by inducing only mild symptoms on zucchini and wax gourd while providing cross-protection against the homologous severe virus. However, their genetic stability remains to be determined through long-term serial passages to ensure the absence of reversion to severe phenotypes. Since ZTMV is known to be transmitted by aphids [50], further investigation is needed to determine whether these mild strains are aphid-transmissible. However, the TuMV mild strains indicated that the lower RNA-silencing suppression function of HC-Pro also results in loss of the aphid transmission ability [34].

In this study, Indirect ELISA was used to measure the relative viral titer for individual ZTMV wild-type and mutants in plant. However, the inhibitory substances in crude plant extracts can potentially interfere with the enzymatic reaction, a phenomenon known as the matrix effect, which may lead to an underestimation of viral concentration. To mitigate this risk, all samples were uniformly diluted at 1:200 ratio, a regularly used practice in plant virological assays [24,26,34]. Furthermore, our preliminary optimization indicated that this dilution factor ensured that the observed differences in absorbance reflect real symptoms in virus-infected plant trends in viral accumulation. While end-point dilution remains the definitive method for absolute quantification, the relative absorbance values provided by indirect ELISA here offer a robust and reproducible comparison suitable for the objectives of this research.

ZTMV has been reported in several regions worldwide and infects multiple cucurbit crops [2,51,52,53]. In Taiwan, ZTMV is an emerging virus, currently documented only on wax gourd [3,4]. To prevent its further spread, early implementation of disease management strategies is essential. Taken together, several attenuated ZTMV strains were successfully developed, exhibiting strong cross-protection effects against ZTMV infection in both zucchini and wax gourd under greenhouse conditions. For practical application, field trials will be required to validate their protective efficacy under natural conditions. Moreover, potential interactions with other cucurbit viruses, either synergistic or protective, should be further investigated in future studies.

## Figures and Tables

**Table 1:** Cross-protection efficacy of zucchini tigre mosaic virus (ZTMV) mild mutants against ZTMV-TW and recombinant ZTMV-GFP on zucchini and wax gourd plants.

Inoculated Mild Strains ^a^	Zucchini	Wax Gourd	
	ZTMV-GFP ^b^	ZTMV-GFP	ZTMV-TW ^b^
F7I	ND ^c^	100% (20/20)	69% (24/35)
R181I	100% (35/35) ^d^	100% (20/20)	17% (6/35)
D397N	ND	100% (20/20)	100% (35/35)
R181I+D397N	100% (35/35)	50% (10/20)	8% (3/35)
F206L+D397N	ND	100% (20/20)	94% (33/35)
Mock	0% (0/15)	0% (0/10)	0% (0/15)

^a^ Zucchini (*Cucurbita pepo* var. *cylindrica*) and wax gourd (*Benincasa hispida*) plants were inoculated with mild mutants and then challenged with severe strains 15 days after the protective inoculation. ^b^ ZTMV-TW is Taiwan isolate of ZTMV, which is a severe strain; ZTMV-GFP is a recombinant of ZTMV-TW containing GFP. ^c^ ND indicates not tested as the mild strains induced severe symptoms. ^d^ Total number of protected/total number of challenged in three independent experiments.

## Data Availability

The data presented in this study are available from the corresponding author on request.

## Supplementary Materials

The following supporting information can be downloaded at https://www.mdpi.com/article/10.3390/v18040411/s1, Figure S1: Construction of the in vivo infectious clone of zucchini tigre mosaic virus Taiwan isolate (p35S ZTMV-TW); Figure S2: Schematic representation of the mutation positions in the HC-Pro gene of the zucchini tigre mosaic virus Taiwan isolate (ZTMV-TW); Figure S3: The cross-protection effectiveness of the zucchini tigre mosaic virus (ZTMV) HC-Pro mutated mild viruses F7I, R181I, D397N, R181I+D397N, and F206L+D397N against wild-type ZTMV-TW (WT) on wax gourd (*Benincasa hispida*) plants; Figure S4: Prediction of protein 3D structure of potyvirus HC-Pros by Alphafold3; Table S1: Primers used in this study.

## References

[1] Lecoq H., Katis N. (2014). Control of cucurbit viruses. Adv. Virus Res..

[2] Peng B., Liu L., Wu H., Kang B., Fei Z., Gu Q. (2021). Interspecific Recombination Between Zucchini Tigre Mosaic Virus and Papaya Ringspot Virus Infecting Cucurbits in China. Front. Microbiol..

[3] Chen Y.-K., Shih P.-J., Chao H.-Y. (2023). First report of mosaic disease associated with zucchini tigre mosaic virus in wax gourd (*Benincasa hispida*) in Taiwan. Plant Disease.

[4] Chen Y.-K., Shih P.-J., Chao H.-Y., Huang C.-H. (2025). Characterization of zucchini tigre mosaic virus wax gourd isolate (ZTMV) and development of differentiating methods for identifying ZTMV and papaya ringspot virus (PRSV). J. Plant Med..

[5] Dong Z.X., Lin C.C., Chen Y.K., Chou C.C., Chen T.C. (2022). Identification of an emerging cucumber virus in Taiwan using Oxford nanopore sequencing technology. Plant Methods.

[6] Thompson J.R., Tepfer M. (2010). Assessment of the benefits and risks for engineered virus resistance. Adv. Virus Res..

[7] McKinney H. (1929). Mosaic diseases in the Canary Islands, West Africa and Gibraltar. J. Agric. Res..

[8] Matthews R.E.F., Hull R. (2002). Matthews’ Plant Virology.

[9] Lin S.-S., Wu H.-W., Jan F.-J., Hou R.F., Yeh S.-D. (2007). Modifications of the helper component-protease of Zucchini yellow mosaic virus for generation of attenuated mutants for cross protection against severe infection. Phytopathology.

[10] Kung Y.-J., Lin P.-C., Yeh S.-D., Hong S.-F., Chua N.-H., Liu L.-Y., Lin C.-P., Huang Y.-H., Wu H.-W., Chen C.-C. (2014). Genetic analyses of the FRNK motif function of Turnip mosaic virus uncover multiple and potentially interactive pathways of cross-protection. Mol. Plant Microbe Interact..

[11] Carr J., Loebenstein G. (2010). Natural and Engineered Resistance to Plant Viruses: Part II.

[12] Lecoq H., Lemaire J.-M., Wipf-Scheibel C. (1991). Control of zucchini yellow mosaic virus in squash by cross protection. Plant Dis..

[13] Wang H., Gonsalves D., Provvidenti R., Lecoq H. (1991). Effectiveness of cross protection by a mild strain of zucchini yellow mosaic virus in cucumber, melon, and squash. Plant Dis..

[14] Yeh S.-D., Gonsalves D. (1984). Evaluation of induced mutants of papaya ringspot virus for control by cross protection. Phytopathology.

[15] Wang H., Yeh S., Chiu R., Gonsalves D. (1987). Effectiveness of cross-protection by mild mutants of papaya ringspot virus for control of ringspot disease of papaya in Taiwan. Plant Dis..

[16] Yeh S.-D., Gonsalves D. (1994). Practices and perspective of control of papaya ringspot virus by cross protection. Advances in Disease Vector Research.

[17] Tran T.T.Y., Cheng H.W., Nguyen V., Yeh S.D. (2023). Modification of the Helper Component Proteinase of Papaya Ringspot Virus Vietnam Isolate to Generate Attenuated Mutants for Disease Management by Cross Protection. Phytopathology.

[18] Cheng H.W., Lin T.T., Huang C.H., Raja J.A.J., Yeh S.D. (2023). Modification of Papaya Ringspot Virus HC-Pro to Generate Effective Attenuated Mutants for Overcoming the Problem of Strain-Specific Cross Protection. Plant Disease.

[19] Tran T.T.Y., Lin T.T., Chang C.P., Chen C.H., Nguyen V., Yeh S.D. (2022). Generation of Mild Recombinants of Papaya Ringspot Virus to Minimize the Problem of Strain-Specific Cross-Protection. Phytopathology.

[20] Gaba V., Zelcer A., Gal-On A. (2004). Cucurbit biotechnology-the importance of virus resistance. In Vitro Cell Dev. Biol. Plant.

[21] Demler S., Rucker D., De Zoeten G., Ziegler A., Robinson D., Murant A. (1996). The satellite RNAs associated with the groundnut rosette disease complex and pea enation mosaic virus: Sequence similarities and ability of each other’s helper virus to support their replication. J. Gen. Virol..

[22] Perring T.M., Farrar C.A., Blua M.J., Wang H., Gonsalves D. (1995). Cross protection of cantaloupe with a mild strain of zucchini yellow mosaic virus: Effectiveness and application. Crop Prot..

[23] Cho J., Ullman D., Wheatley E., Holly J., Gonsalves D. (1992). Commercialization of ZYMV cross protection for zucchini production in Hawaii. Phytopathology.

[24] Do D.H., Ngo X.T., Yeh S.D. (2024). The Generation of Attenuated Mutants of East Asian Passiflora Virus via Deletion and Mutation in the N-Terminal Region of the HC-Pro Gene for Control through Cross-Protection. Viruses.

[25] Chong Y.H., Do D.H., Cheng H.W., Raja J.A.J., Ngo X.T., Hwang S.G., Yeh S.D. (2023). Generation of Attenuated Mutants of East Asian Passiflora Virus for Disease Management by Cross Protection. Mol. Plant Microbe Interact..

[26] Do D.H., Nguyen T.B., Ha V.C., Raja J.A.J., Yeh S.D. (2023). Generation of Attenuated Passiflora Mottle Virus Through Modification of the Helper Component Protease for Cross Protection. Phytopathology.

[27] Yarden G., Hemo R., Livne H., Maoz E., Lev E., Lecoq H., Raccah B. (2000). Cross-protection of cucurbitaceae from zucchini yellow mosaic potyvirus. Proceedings of the VII Eucarpia Meeting on Cucurbit Genetics and Breeding 510.

[28] Valli A., Gallo A., Calvo M., Pérez J.d.J., García J.A. (2014). A novel role of the potyviral helper component proteinase contributes to enhance the yield of viral particles. J. Virol..

[29] Peng Y.-h., Kadoury D., Gal-On A., Huet H., Wang Y., Raccah B. (1998). Mutations in the HC-Pro gene of zucchini yellow mosaic potyvirus: Effects on aphid transmission and binding to purified virions. J. Gen. Virol..

[30] Carrington J.C., Freed D.D., Sanders T.C. (1989). Autocatalytic processing of the potyvirus helper component proteinase in *Escherichia coli* and in vitro. J. Virol..

[31] Torres-Barcelo C., Martin S., Daros J.-A., Elena S.F. (2008). From hypo-to hypersuppression: Effect of amino acid substitutions on the RNA-silencing suppressor activity of the Tobacco etch potyvirus HC-Pro. Genetics.

[32] Urcuqui-Inchima S., Haenni A.-L., Bernardi F. (2001). Potyvirus proteins: A wealth of functions. Virus Res..

[33] Gal-On A., Katsir P., Yongzang W. (2000). Genetic engineering of attenuated viral cDNA of Zucchini yellow mosaic virus for protection of cucurbits. Proceedings of the VII Eucarpia Meeting on Cucurbit Genetics and Breeding 510.

[34] Raja J.A.J., Huang C.H., Chen C.C., Hu W.C., Cheng H.W., Goh R.P., Chao C.H., Tan Y.R., Yeh S.D. (2022). Modification of the N-terminal FWKG-alphaH1 element of potyviral HC-Pro affects its multiple functions and generates effective attenuated mutants for cross-protection. Mol. Plant Pathol..

[35] Tuo D., Zhou P., Zhao G., Yan P., Tan D., Li X., Shen W. (2020). A Double Mutation in the Conserved Motifs of the Helper Component Protease of Papaya Leaf Distortion Mosaic Virus for the Generation of a Cross-Protective Attenuated Strain. Phytopathology.

[36] Reynolds E.S. (1963). The use of lead citrate at high pH as an electron-opaque stain in electron microscopy. J. Cell Biol..

[37] Engvall E., Perlmann P. (1971). Enzyme-linked immunosorbent assay (ELISA) quantitative assay of immunoglobulin G. Immunochemistry.

[38] Abramson J., Adler J., Dunger J., Evans R., Green T., Pritzel A., Ronneberger O., Willmore L., Ballard A.J., Bambrick J. (2024). Addendum: Accurate structure prediction of biomolecular interactions with AlphaFold 3. Nature.

[39] Goddard T.D., Huang C.C., Meng E.C., Pettersen E.F., Couch G.S., Morris J.H., Ferrin T.E. (2018). UCSF ChimeraX: Meeting modern challenges in visualization and analysis. Protein Sci..

[40] Chiang C.-H., Lee C.-Y., Wang C.-H., Jan F.-J., Lin S.-S., Chen T.-C., Raja J.A., Yeh S.-D. (2007). Genetic analysis of an attenuated Papaya ringspot virus strain applied for cross-protection. Eur. J. Plant Pathol..

[41] Tran T.N., Cheng H.W., Xie X.Y., Raja J.A.J., Yeh S.D. (2023). Concurrent Control of Two Aphid-Borne Potyviruses in Cucurbits by Two-in-One Vaccine. Phytopathology.

[42] Shiboleth Y.M., Haronsky E., Leibman D., Arazi T., Wassenegger M., Whitham S.A., Gaba V., Gal-On A. (2007). The conserved FRNK box in HC-Pro, a plant viral suppressor of gene silencing, is required for small RNA binding and mediates symptom development. J. Virol..

[43] German-Retana S., Candresse T., Alias E., Delbos R.-P., Le Gall O. (2000). Effects of green fluorescent protein or β-glucuronidase tagging on the accumulation and pathogenicity of a resistance-breaking Lettuce mosaic virus isolate in susceptible and resistant lettuce cultivars. Mol. Plant Microbe Interact..

[44] Johansen I.E., Lund O.S., Hjulsager C.K., Laursen J. (2001). Recessive resistance in Pisum sativum and potyvirus pathotype resolved in a gene-for-cistron correspondence between host and virus. J. Virol..

[45] Dietrich C., Maiss E. (2003). Fluorescent labelling reveals spatial separation of potyvirus populations in mixed infected Nicotiana benthamiana plants. J. Gen. Virol..

[46] Ivanov K.I., Puustinen P., Gabrenaite R., Vihinen H., R önnstrand L., Valmu L., Kalkkinen N., Mäkinen K. (2003). Phosphorylation of the potyvirus capsid protein by protein kinase CK2 and its relevance for virus infection. Plant Cell.

[47] Choi I.R., Stenger D.C., Morris T.J., French R. (2000). A plant virus vector for systemic expression of foreign genes in cereals. Plant J..

[48] Arazi T., Slutsky S.G., Shiboleth Y.M., Wang Y., Rubinstein M., Barak S., Yang J., Gal-On A. (2001). Engineering zucchini yellow mosaic potyvirus as a non-pathogenic vector for expression of heterologous proteins in cucurbits. J. Biotechnol..

[49] Chen C.-C., Chen T.-C., Raja J.A., Chang C.-A., Chen L.-W., Lin S.-S., Yeh S.-D. (2007). Effectiveness and stability of heterologous proteins expressed in plants by Turnip mosaic virus vector at five different insertion sites. Virus Res..

[50] Quiot-Douine L., Purcifull D., Hiebert E., De Mejia M. (1986). Serological relationships and in vitro translation of an antigenically distinct strain of papaya ringspot virus. Phytopathology.

[51] Romay G., Lecoq H., Desbiez C. (2014). Zucchini tigré mosaic virus is a distinct potyvirus in the papaya ringspot virus cluster: Molecular and biological insights. Arch. Virol..

[52] Zhou C.J., Liang Z.R., Zhang J.B., Huang B., He G.Z., Zhong C.N., Ouyang T.X. (2023). First Report of Zucchini Tigre Mosaic Virus Infecting Four Cucurbit Crops in China. Plant Disease.

[53] Zhu L.J., Xing J., Li J., Lin W., Chi Y., Su J., Zhang J., Xu Z. (2025). Virome Analysis Deciphers the First Virus Occurrence in *Melothria scabra*, Revealing Two Potyviruses, Including a Highly Divergent Zucchini Yellow Mmosaic Virus Isolate. Plant Pathol. J..

