# Supplementary material for: Generation of Zucchini Tigre Mosaic Virus Mild Strains for Application in Cross-Protection
Source: Viruses. 2026 Mar 26;18(4):411. doi: 10.3390/v18040411 (PMC13119878; doi:10.3390/v18040411)
Supplement: Supplementary file 1 [file viruses-18-00411-s001.zip › viruses-4196421-supplementary.pdf]

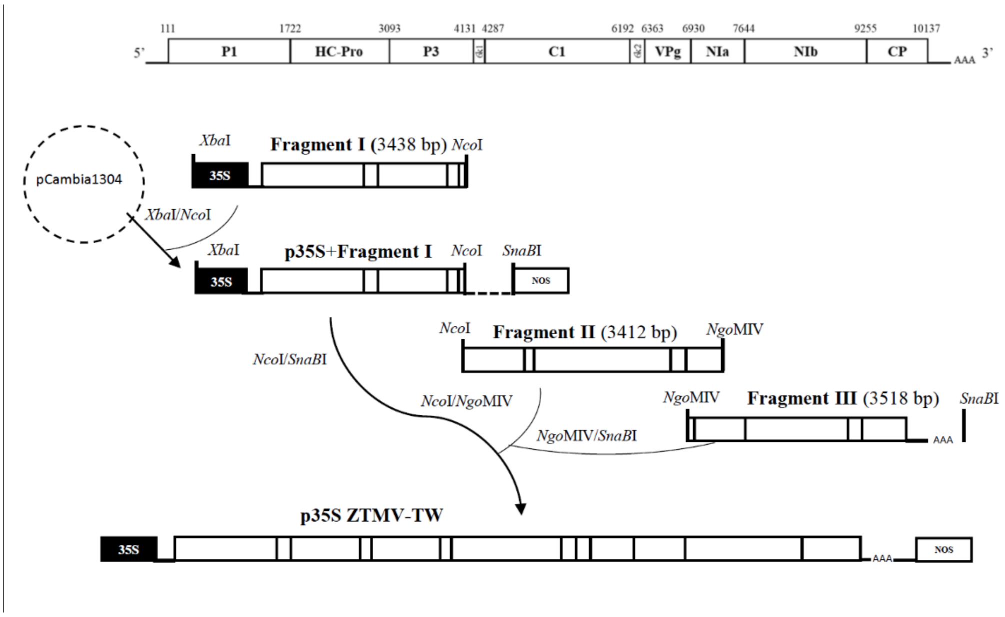

Figure S1. Construction of the *in vivo* infectious clone of zucchini tigre mosaic virus Taiwan isolate (p35S ZTMV-TW). Three pairs of ZTMV-specific primers were used to amplify viral sequence, fragment I、 II and III, by RT-PCR. The 35S promoter fragment was linked at the N-terminus of fragment I through overlapping PCR. The “p35S+fragment I” was digested with *Xba*I and *Nco*I restriction enzymes and ligated into the pCambia 1340 agrobacteria binary vector. Then, the fragment II and fragment III were digested with *Nco*I, *NgoM*IV, and *SnaB*I restriction enzymes and ligated into the pCambia-ZTMV-fragment I. The constructed plasmid containing the complete genome sequence of ZTMV-TW was designated as ZTMV infectious clone (p35S ZTMV-TW).

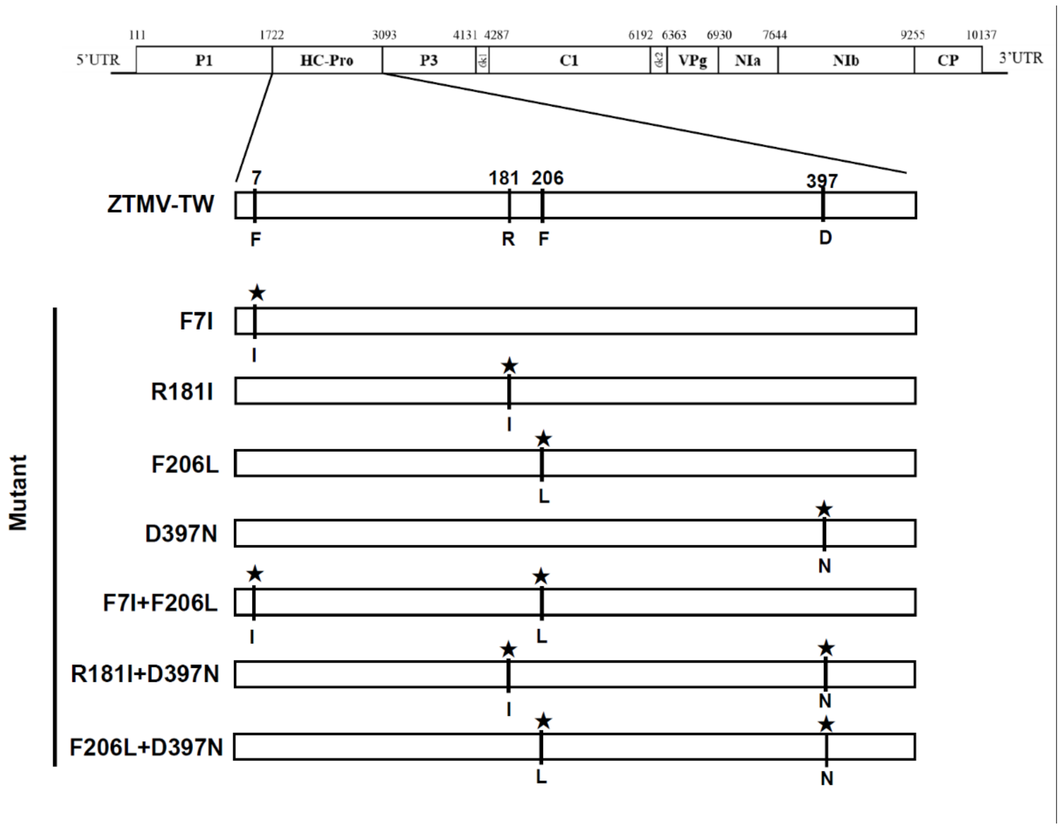

Figure S2. Schematic representation of the mutation positions in the HC-Pro gene of the zucchini tigre mosaic virus Taiwan isolate (ZTMV-TW). Bar (|), asterisk (★), and characters indicate the position and residue of amino acids in the HC-Pro gene of the wild type ZTMV-TW, while the asterisks are mutations on the constructed ZTMV mutants, including F7I, R181I, F206L, D397N, F7I+F206L, R181I+D397N, and F206L+D397N, respectively.

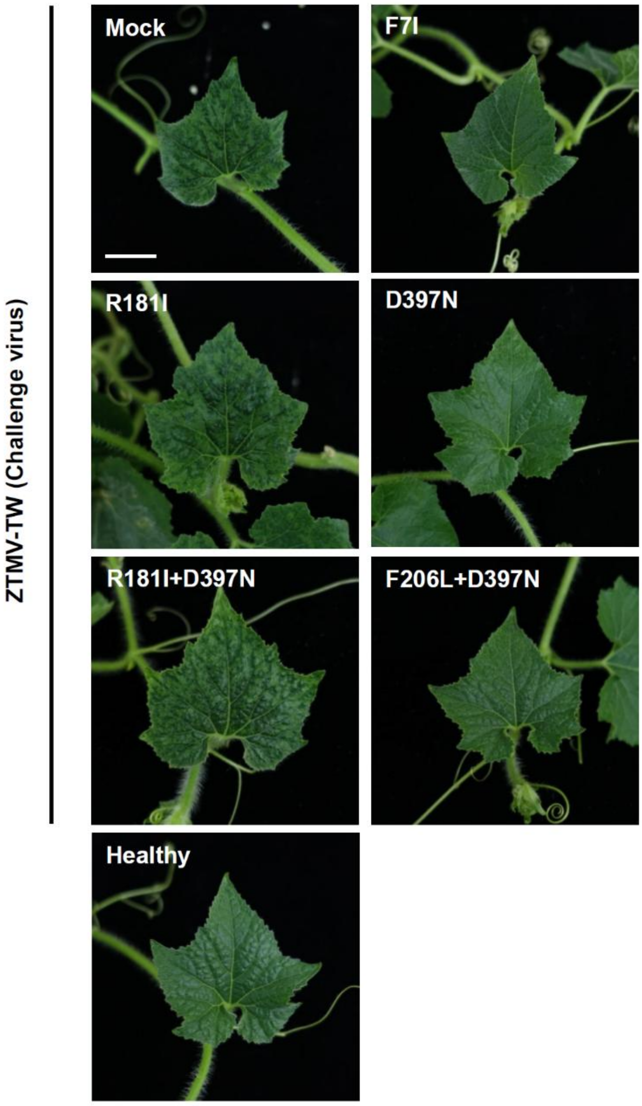

Figure S3. The cross-protection effectiveness of the zucchini tigre mosaic virus (ZTMV) HC-Pro mutated mild viruses F7I, R181I, D397N, R181I+D397N, and F206L+D397N against wild type ZTMV-TW (WT) on wax gourd (*Benincasa hispida*) plants. All plants were challenged with wild type ZTMV-TW after 15 days of protective virus inoculation. Symptoms were photographed at 14 days after the challenge virus inoculation. Buffer-inoculated plants (Mock) and un-inoculation plants (Healthy) were used as negative control for cross protection and no virus infection control, respectively. Scale bar: 3 cm.

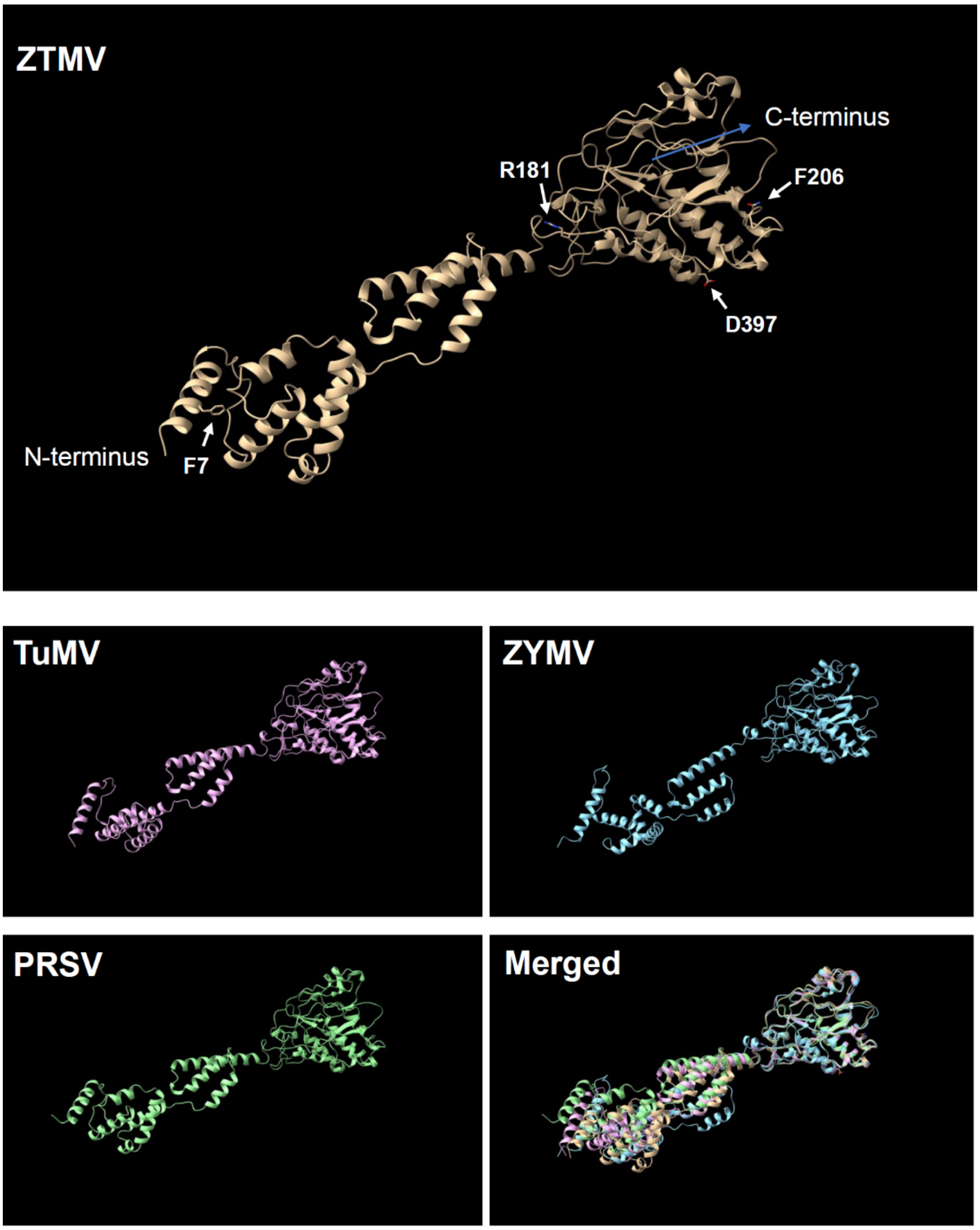

Figure S4. Prediction of protein 3D structure of potyvirus HC-Pros by Alphafold3. The predicted potyviral HC-Pro protein models are displayed individually and in a merged view. The color-coding and pTM are as follows: Brown/0.68 (ZTMV), Pink/0.68 (TuMV), Blue/0.66 (ZYMV), and Green/0.69 (PRSV). White arrows indicate the side chain group of amino acid positions subjected to mutations in this study. A blue arrow indicates the C-terminus of ZTMV HC-Pro protein.

Table S1. Primers used in this study

Name	Sequence 5'-3'	Underline notes
**Cloning primers**		
pC-p35S-F	GGGGATCCTCTAGAGAGACTTTTC	*Xba*I
pC-p35S-OE-R	GATTTGTTTTATTTTGTTCTCTCCAA	
pC-ZTMV-OE-F	TTGGAGAGAACAAAATAAAACAAATC	
pC-ZTMV-1R	ATGAAATCCATGGCCTTGAA	*Nco*I
pC-ZTMV-2F	AGAAATAATGGAAAGAGGTTCAAGG	
pV-ZTMV-II R	TCTTAGTTCAAATTCGCGCTCTGGA	
pV-ZTMV-II F	TTGTGACAACAAACTCACGA	
*Sna*BI-Oligo-dT	AACGGTGATACGTATTTTTTTTTTTTTTTTTTTTT TTTTTTTT	*SnaB*I
GFP-1R	CTCCTTTACTACTGTAGTGCTCCAT	
GFP-2F	GCACTACAGTAGTAAAGGAGAAGAA	
GFP HC-3R	CGATTGGTAAAACACATTTTTGTATAGTTCA	
GFP HC-4F	TGTGTTTTACCAATCGAGTGATGTTGCTGAG	
**Site direct mutagenesis primers**		
RHC F7I	AAGCCAAA**T**TTTCTCAGCAACATCA	Phe→Ile
FHC F7I	GAAA**A**TTTGGCTTGGTTTTAATAGAGCG	
RHC R181I *Afe*I	GCTTGTTT**A**TGAAACTTTCAACACTTCC	Arg→Ile
FHC R181I *Afe*I	GTTTCA**T**AAACAAGCG**C**TCAGGCAA	+*Afe*I
RHC F206L	CCATAGGA**G**ATTTCCATTCCTATCCAA	Phe→Leu
FHC F206L	GGAAAT**C**TCCTATGGGGTGAACGTCAATAT	
RHC D397N	CTTGCTGCGT**T**CGGGTGAAATATGA	Asp→Asn
FHC D397N	CG**A**ACGCAGCAAGTGCTGAACTACC	
**Detection primers**		
fZTMV-F	AGCRTGTGGYAHCC	
gourd-R	TCCCACCAYTTYTCRAAHGT	

*, Underlines indicate restriction enzyme, "+" indicates the created restriction sites. Bold characters indicate the modified nucleotides. Double underlines indicate the amino acid alternating nucleotides.
